# Estimating the potential for dementia prevention through modifiable risk factors elimination in the real-world setting: a population-based study

**DOI:** 10.1186/s13195-020-00661-y

**Published:** 2020-08-07

**Authors:** Elena Rolandi, Daniele Zaccaria, Roberta Vaccaro, Simona Abbondanza, Laura Pettinato, Annalisa Davin, Antonio Guaita

**Affiliations:** 1 “Golgi Cenci” Foundation, Corso San Martino 10, 20081 Abbiategrasso, Italy; 2 Department of Business Economics, Health and Social Care Centre of Competence on Ageing, University of Applied Sciences and Arts of Southern Switzerland, Stabile Piazzetta, Via Violino 11, CH-6928 Manno, Switzerland

**Keywords:** Dementia, Alzheimer’s disease, Modifiable risk factors, Dementia prevention, Public health, Population attributable fraction, Aging

## Abstract

**Background:**

Preventing dementia onset is one of the global public health priorities: around 35% of dementia cases could be attributable to modifiable risk factors. These estimates relied on secondary data and did not consider the concurrent effect of non-modifiable factors and death.

Here, we aimed to estimate the potential reduction of dementia incidence due to modifiable risk factors elimination, controlling for non-modifiable risk factors and for the competing risk of death.

**Methods:**

Participants from the InveCe.Ab population-based prospective cohort (Abbiategrasso, Italy) without a baseline dementia diagnosis and attending at least one follow-up visit were included (*N* = 1100). Participants underwent multidimensional assessment at baseline and after 2, 4, and 8 years, from November 2009 to January 2019.

Modifiable risk factors were low education, obesity, hypertension, diabetes, depression, smoking, physical inactivity, hearing loss, loneliness, heart disease, stroke, head injury, and delirium. Non-modifiable risk factors were age, sex, and APOE ε4 genotype. The primary endpoint was dementia diagnosis within the follow-up period (DSM-IV criteria). We performed competing risk regression models to obtain sub-hazard ratio (SHR) for each exposure, with death as competing risk. The exposures associated with dementia were included in a multivariable model to estimate their independent influence on dementia and the corresponding population attributable fraction (PAF).

**Results:**

Within the study period (mean follow-up, 82.3 months), 111 participants developed dementia (10.1%). In the multivariable model, APOE ε4 (SHR = 1.89, 95% CI 1.22–2.92, *p* = 0.005), diabetes (SHR = 1.56, 95% CI 1.00–2.39, *p* = 0.043), heart disease (SHR = 1.56, 95% CI 1.03–2.36, *p* = 0.037), stroke (SHR = 2.31, 95% CI 1.35–3.95, *p* = 0.002), and delirium (SHR = 8.70, 95% CI 3.26–23.24, *p* <  0.001) were independently associated with increased dementia risk. In the present cohort, around 40% of dementia cases could be attributable to preventable comorbid diseases.

**Conclusions:**

APOE ε4, diabetes, heart disease, stroke, and delirium independently increased the risk of late-life dementia, controlling for the competing risk of death. Preventive intervention addressed to these clinical populations could be an effective approach to reduce dementia incidence. Further studies on different population-based cohort are needed to obtain more generalizable findings of the potential of dementia prevention in the real-world setting.

**Trial registration:**

ClinicalTrials.gov, NCT01345110.

## Background

Late-onset dementia is a multi-factorial disorder causing huge economic and social costs for public health and families. Advancing age remains one of the main risk factors for dementia: from 65 years, dementia prevalence nearly doubled every 5 years [1]. Thus, in absence of any effective treatment and considering the worldwide increase in life expectancy, the global prevalence of dementia is expected to constantly grow in the next decades [2]. In this view, preventing or delaying dementia onset is one of the global public health priorities. The Lancet International Commission on Dementia Prevention, Intervention and Care points out that around 35% of dementia cases could be prevented targeting 9 modifiable risk factors in different life periods: low education in early life, hearing loss, hypertension and obesity in midlife, smoking, depression, physical inactivity, diabetes, and social isolation in late-life [3].

Beyond these cardiovascular and lifestyle factors, other preventable medical conditions increased dementia risk, such as stroke [4], delirium [5], heart disease [6], and traumatic brain injury [7].

Moreover, non-modifiable factors, such as age and APOE ε4, significantly increase the risk of dementia and should be taken into account. Another important confounding which is rarely considered in disease outcome research is the concurrent risk of death, which is particularly relevant in older population and, if not considered, may lead to biased estimation of risk [8].

The InveCe.Ab study (Invecchiamento Cerebrale in Abbiategrasso, i.e., Brain aging in Abbiategrasso; ClinicalTrials.gov, NCT01345110) is a population-based study aimed to estimate dementia incidence and to explore socio-demographic, clinical, and lifestyle factors associated with aging and dementia [9]. The InveCe.Ab participants underwent multidimensional evaluations across 8 years, thus allowing both the collection of a wide range of risk factors at baseline and the prospective detection of dementia onset within the study period.

The present study aimed to estimate the potential reduction of dementia incidence due to modifiable risk factors elimination, taking into account the concurrent effect of non-modifiable risk factors and the competing risk of death. This approach would provide a more naturalistic picture of the potential of dementia prevention in the real-world setting, based on primary data from a population-based prospective cohort study and concurrently considering almost all the known risk factors.

## Methods

### Participants

The InveCe.Ab study is a single-step multidimensional population-based study, which comprised both a cross-sectional and a longitudinal phase. The eligible population consisted of all the 1773 people born between 1935 and 1939 and residing in Abbiategrasso (Milan, Italy) on the prevalence day (November 1, 2009). This age range (70–74 years) is considered a transitional age between late adulthood and old age in which persons usually notice the first cognitive changes leading to medical help seeking, but are still responsive to social, cardiovascular and lifestyle protective factors [9]. The study design is described in details elsewhere [9]. Briefly, 1321 older adults agreed to participate in the study and underwent a multidimensional assessment. Enrolled participants were re-contacted after 2, 4, and 8 years to perform follow-up evaluations, using the same assessment procedures of the baseline ones. Participants were eligible for the present investigation if they completed the baseline neuropsychological and medical assessments, confirming the absence of dementia, mental retardation, or psychosis, and attended at least one follow-up visit across 8 years.

All the participants provided written informed consent to the study procedures, which were in accordance with the Declaration of Helsinki. The study protocol was approved by the Ethics Committee of the University of Pavia on October 6, 2009 (Committee report 3/2009).

### Multidimensional assessment

The multidimensional assessment consisted of blood sampling, social and lifestyle interview, geriatric visit, and neuropsychological assessment.

During the social and lifestyle interview, trained personnel help participants to compile self-report questionnaires on socio-demographics features and lifestyle habits.

The geriatricians collected a guided and detailed medical history, recorded current medications, and performed physical examination with special attention to neurologic signs and symptoms. Participants were further asked to bring medical reports of ambulatory visits and hospitalizations occurred in the period preceding the visit.

The neuropsychologists investigated the presence of the DSM-IV-TR (Diagnostic and Statistical Manual of Mental Disorders IV) criteria for major depression and dysthymia through clinical interview and administered the 15-items form of the Geriatric Depression Scale (GDS, [10]). A comprehensive neuropsychological test battery was administered to evaluate global cognition and the main cognitive domains (language, memory, attention, executive functions, visuo-spatial abilities).

All the details on instruments and study procedures are reported in the previously published study protocol [9].

### Risk factors: operational definitions

The main established modifiable and non-modifiable risk factors for dementia were detected at each wave of the InveCe.Ab study. For the aim of the present investigation, baseline exposure to the risk factors was considered. Table 1 summarizes the operational definitions for each risk factor, which are further detailed in the present paragraph.

**Table 1:** Summary of the operational definitions of the modifiable and non-modifiable risk factors

**Non-modifiable**	
▪ **Age**: class 1935–1937	
▪ **Sex**: females	
▪ **APOE**: ε4 carriers	
**Modifiable**	
▪ **Low education**: ≤ 5 years of formal education	
▪ **Obesity**: BMI ≥ 30 kg/m^2^	
▪ **Hypertension**: systolic blood pressure > 130 mmHg	
OR diastolic blood pressure > 80 mmHg	
OR antihypertensive medication	
▪ **Diabetes**: fasting glucose ≥ 126 mg/dL	
OR diabetes treatment	
▪ **Depression**: DSM-IV-TR diagnosis of major depression or dysthymia OR at least 3 of these features: (i) history of depression, (ii) depression treatment, (iii) GDS ≥ 8, and (iv) self-reported depressed mood during the last week.	
▪ **Smoking**: current smokers	
▪ **Physical inactivity**: absence of any weekly vigorous or moderate leisure time physical activity	
▪ **Hearing loss**: evaluated by the physician with the Whispered Voice Test	
▪ **Loneliness**: feeling lonely during the last week	
▪ **Heart disease**: anamnestic diagnosis	
▪ **Stroke**: anamnestic diagnosis	
▪ **Head injury**: anamnestic diagnosis	
▪ **Delirium**: anamnestic diagnosis	

#### Non-modifiable risk factors

The DNA of each participant was extracted from blood sample and analyzed using real-time PCR (Applied Biosystems) to ascertain the presence of the APOE ε4 genotype. As regards age, participants were stratified in two subgroups according to birth cohort (i.e., 1935–1937 versus 1938–1939). No conclusive evidence exists on the effect of sex on dementia incidence; however, several studies showed sex-specific susceptibility to risk factors and different disease manifestation in Alzheimer’s disease [11]; therefore, we considered female sex as a non-modifiable risk factor.

#### Modifiable risk factors

Low education was defined as less or equal than 5 years of formal education attendance, corresponding to primary school completion.

Body mass index (BMI) was calculated with the appropriate formula (kg/m^2^), and obesity was ascertained by a BMI greater or equal to 30.

Hypertension was confirmed if at least one of the following conditions was present: (i) systolic blood pressure greater than 130 mmHg; (ii) diastolic blood pressure greater than 80 mmHg; and (iii) use of antihypertensive medications such as angiotensin-converting-enzyme inhibitor (ACE inhibitor), calcium antagonist, angiotensin-II- receptor antagonists (sartans), diuretics, or beta blockers.

Diabetes was confirmed if at least one of the following conditions was present: (i) fasting glucose greater or equal to 126 mg/dL and (ii) use of insulin or oral hypoglycemic treatment.

Depression was evaluated combining information collected during the medical and neuropsychological assessment, as previously reported [12]. Briefly, clinically significant depression was established if DSM-IV-TR criteria for major depression or dysthymia were fulfilled [13] or if at least 3 of the following features were present: (i) history of depression; (ii) use of antidepressant or anxiolytic/hypnotic drugs; (iii) GDS ≥ 8; and (iv) self-reported depressed mood during the last week.

Smoking and physical activity habits were investigated during the social and lifestyle interview. Current smoking was considered a risk condition. Physical inactivity was defined as the absence of any weekly leisure time physical activity.

The physician clinically evaluated hearing ability with the Whispered Voice Test, in which the examiner stands behind the seated patient and whispers a standardized combination of six numbers and letters. Hearing loss was ascertained if participants repeated less than 3 items [14].

Self-perceived loneliness was investigated by the following yes/no single question asked by the physician: “Have you felt lonely/abandoned often during the last week?” [15, 16].

Finally, the physicians investigated the anamnestic diagnoses of heart disease (myocardial infarction, cardiac dysrhythmias, heart failure, angina pectoris, coronary bypass), stroke, head injury and delirium (medical history based on DSM-IV-TR criteria [13]).

### Outcome: dementia diagnosis

The primary endpoint for the present study was cumulative incidence of all-cause dementia within the study period. At each evaluation wave, after medical and neuropsychological assessment completion, an expert geriatrician (A.G.) confirmed the presence/absence of dementia, based on the DSM IV-TR criteria for dementia syndrome. If the diagnosis was firstly formulated after the study visits, dementia onset was set at the date of the corresponding evaluation wave. Alternatively, if a dementia diagnosis was already made during the period between consecutive study visits, the date of the diagnosis formulation was retrospectively collected.

### Statistical analysis

All the statistical analyses were done using Stata release 15 (StataCorp 2017). Firstly, using a Pearson’s Chi-square test, we compared the proportion of non-modifiable and modifiable risk factors exposure between participants who developed dementia over the follow-up period with those who did not. We considered statistically significant difference if *p* value is equal or less than 0.050. Then, assuming that death has to be considered an event that precludes the occurrence of our event of interest, that is diagnosis of dementia, we performed competing risk models, with death as a competitive event [17–19]. Considering the prediction nature of our main research aim, we fitted univariable Fine and Gray’s semiparametric proportional sub-distribution hazards models to obtain sub-hazard ratios (SHRs), with 95% CI, representing the effect of each risk factor on the cumulative incidence function (CIF) of dementia and death in a competing risk situation [20–22]. We considered time in months from the baseline evaluation as a basic timescale variable, and date of first evaluation as the time origin. The risk factors found to have a significant sub-hazard ratio at level *p* <  0.150 in univariable models regarding the event of interest (dementia) were included in the competing risks multivariable model, similarly to what has been done by Ritchie and colleagues [23]. The assumption of proportionality of hazards for both univariable and multivariable models were assessed testing statistical significance of interaction terms involving failure time. We used the default function of time *g*(*t*) = *t* (see Additional file 1 for details).

Finally, we calculated the population attributable fraction (PAF) with 95% CI for each risk factor considered in the multivariable model, which estimates the proportion of incident cases in a given period that would be prevented if the exposure was entirely eliminated. It takes into account both the relative risk and the frequency of the risk factor in the population studied. In our case, PAF means proportion in risk reduction over 8 years that would be obtained if a specific exposure was entirely eliminated from the InveCe.Ab cohort, while the distribution of other risk factors included in the model remains unchanged. PAF is calculated subtracting population unattributable fraction (PUF), and its confidence limits, from 1. In this way, we compared a hypothetic scenario in which no one is exposed to the given risk factor and the world in which the data were collected. PAF is calculated using the “punafcc” command in Stata [24].

## Results

**Fig. 1:**
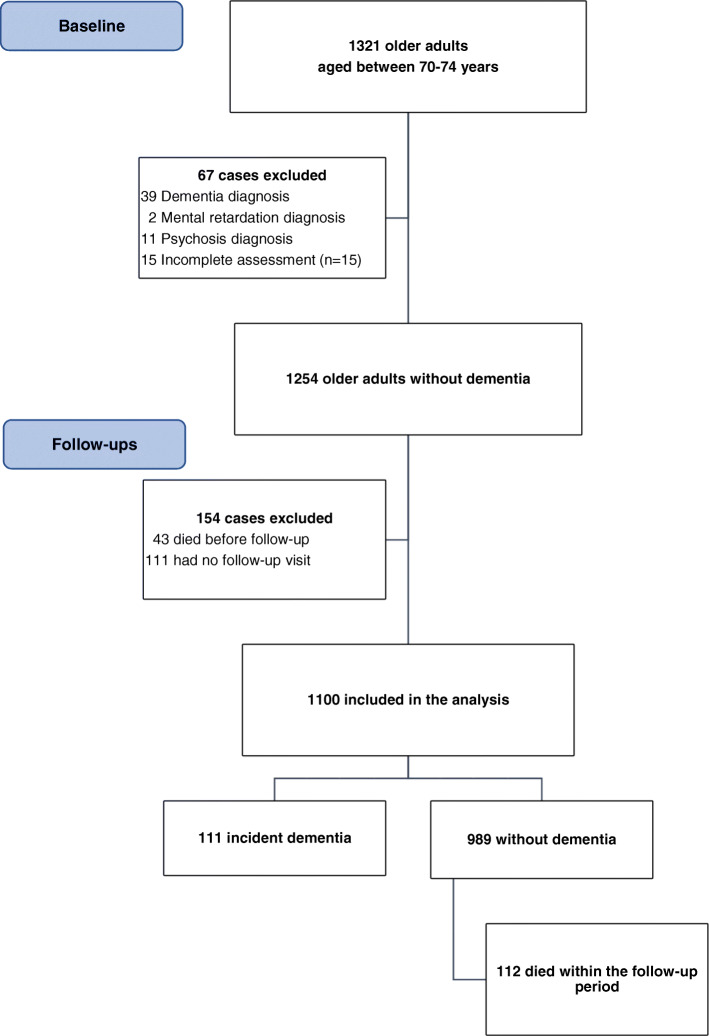
Flow-chart of case selection

Figure 1 displays the flowchart of case selection. At baseline, 1254 cases met the inclusion criteria. Among them, 154 participants did not complete any follow-up visit across 8 years, resulting in a sample for the present investigation of 1100 older adults without a baseline dementia diagnosis aged between 70 and 74 years. Within the study period, 111 participants developed dementia (10.1%). The mean follow-up period was 82.3 months.

Table 2 reports the non-adjusted comparisons of baseline non-modifiable and modifiable risk factors exposure between participants who developed dementia over the follow-up period with those who did not, with chi-squared test. We found statistically significant differences concerning APOE ε4 and the presence of heart disease, stroke, or delirium.

**Table 2:** Descriptive variables and impact of each factor on dementia incidence across 8 years, based on univariable models, with death as a competing event

Risk factors	Without dementia	Incident dementia	***χ***^**2**^ test	Death (competing event)		Dementia	
	***N*** = 989	***N*** = 111	***P*** value	SHR (95% CI)	***P*** value	SHR (95% CI)	***P*** value
**Non-modifiable**							
Age (class 1935–37)	522 (52.7)	69 (62.2)	*0.060*	1.21 (0.82–1.78)	0.332	1.20 (0.82–1.78)	0.347
Sex (female)	534 (54.0)	55 (49.5)	0.373	0.76 (0.53–1.11)	0.156	0.82 (0.56–1.19)	0.287
APOE ε4	166 (16.8)	34 (30.6)	**< 0.001**	0.92 (0.47–12.16)	0.742	2.00 (1.34–3.01)	**0.001**
**Modifiable**							
Low education	548 (55.4)	69 (62.2)	0.174	1.15 (0.79–1.68)	0.459	1.38 (0.94–2.03)	**0.103**
Obesity	259 (27.3)	29 (27.9)	0.908	0.68 (0.42–1.09)	0.107	1.08 (0.70–1.66)	0.716
Hypertension	685 (69.4)	81 (73.6)	0.359	1.08 (0.72–1.63)	0.697	1.27 (0.83–1.94)	0.270
Diabetes	198 (20.2)	31 (28.2)	*0.053*	1.30 (0.84–12.96)	0.200	1.55 (1.02–2.36)	**0.042**
Depression	51 (5.2)	5 (4.5)	0.767	2.11 (1.07–4.16)	**0.032**	0.99 (0.40–2.45)	0.984
Smoking	86 (8.7)	11 (9.9)	0.669	2.04 (1.24–3.37)	**0.005**	1.12 (0.60–2.09)	0.715
Physical inactivity	462 (46.8)	60 (54.1)	*0.145*	1.27 (0.88–1.84)	0.203	1.42 (0.98–2.06)	**0.064**
Hearing loss	123 (12.5)	16 (14.5)	0.539	1.07 (0.62–1.83)	0.803	1.20 (0.71–2.02)	0.493
Loneliness	117 (11.9)	10 (9.1)	0.388	1.31 (0.77–2.21)	0.316	0.82 (0.42–1.59)	0.562
Heart disease	255 (25.8)	42 (37.8)	**0.007**	1.29 (0.87–1.91)	0.206	1.61 (1.09–2.37)	**0.016**
Stroke	64 (6.5)	20 (18.2)	**< 0.001**	2.05 (1.18–3.55)	**0.011**	2.69 (1.67–4.35)	**< 0.001**
Head injury	77 (7.8)	7 (6.3)	0.576	1.12 (0.55–2.24)	0.757	0.84 (0.39–1.80)	0.649
Delirium	5 (0.5)	7 (6.4)	**< 0.001**	1.52 (0.37–6.28)	0.562	8.52 (3.62–20.05)	**< 0.001**

Values represent counts (percentage)

*SHR* sub-hazard ratio

Table 2 further reports the results of univariable competing risk models, to identify which of the exposures considered affect the cumulative incidence of dementia along the observation period. Sub-hazard ratios were reported both for the event of interest (dementia) and for the competing event (death). APOE ε4, diabetes, heart disease, stroke, and delirium increase dementia incidence at *p* <  0.05. Moreover, a trend toward significance was found for physical inactivity and low education (*p* <  0.150). As regards the competing event, depression, smoking, and stroke significantly increase the cumulative incidence of death within the observation period.

Then, we included all the exposures found to be significant for dementia incidence at level *p* <  0.150 in the previous step, in a multivariable sub-distribution hazards model with death as competing event (Table 3). We found that APOE ε4, diabetes, heart disease, stroke, and delirium, independently considered, produce a significant increase in the cumulative incidence of dementia. With regard to the competing event, diabetes and stroke independently increase the cumulative incidence of death.

**Table 3:** Independent influence of each risk factor on dementia incidence across 8 years, based on competing risk multivariable model

Risk factors	Death (competing event)		Dementia		
	SHR (95% CI)	***P*** value	SHR (95% CI)	***P*** value	% PAF (95% CI)
APOE ε4	0.93 (0.57–1.51)	0.761	1.89 (1.22–2.92)	**0.005**	
Low education	1.18 (0.80–1.73)	0.803	1.42 (0.95–2.11)	0.087	18.5 (1.3–23.4)
Diabetes	1.24 (0.80–1.91)	**0.043**	1.56 (1.00–2.39)	**0.043**	9.9 (1.9–17.3)
Physical inactivity	1.22 (0.84–1.78)	0.297	1.33 (0.90–1.96)	0.152	13.3 (3.9–27.7)
Heart disease	1.26 (0.85–1.86)	0.849	1.56 (1.03–2.36)	**0.037**	13.6 (2.8–23.2)
Stroke	2.01 (1.16–3.47)	**0.012**	2.31 (1.35–3.95)	**0.002**	10 (5.8–14.0)
Delirium	1.50 (0.34–6.51)	0.590	8.70 (3.26–23.24)	**< 0.001**	5.7 (5.0–6.5)

*SHR* sub-hazard ratio, *PAF* population attributable fraction

Finally, PAF estimations are reported, which showed the portion of dementia cases independently attributable to each modifiable risk factor, ranging from 5.7% for delirium to 13.6% for heart disease.

## Discussion

In the present study, we aimed to evaluate the potential impact of dementia prevention through risk reduction in the real-world setting, using primary data from a population-based prospective cohort of older adults aged 70–74 years and controlling for non-modifiable factors and for the competing risk of death. Our findings expand and complement current knowledge on the potential impact of dementia prevention by including, beyond the nine modifiable risk factors acknowledged by the Lancet Commission, other preventable medical conditions associated with dementia, namely heart disease, stroke, head injury, and delirium. Among all the risk factors considered, APOE ε4, diabetes, heart disease, stroke and delirium independently increased dementia incidence in late life. Overall, excluding the genetic risk that is not preventable, about 40% of dementia cases in the present population could be attributable to preventable comorbid diseases based on PAF estimations. Noteworthy, diabetes, stroke, heart disease, and delirium, which are also known to be associated with increased mortality [25–27], showed a significant and independent influence on dementia onset, even if death occurrence was controlled. These findings highlight the importance to address preventive initiative to these frail populations, already facing complex clinical needs. In terms of public health, focused and personalized preventive interventions embedded in the care pathways of these clinical populations could be an effective approach to reduce late-life dementia incidence. Another feasible and complementary approach is to further boost recommendations on cardiovascular health starting from middle age, as a common factor reliably related to several chronic diseases, leading to adverse outcomes and huge burden for families and communities, such as diabetes, stroke, heart disease, and dementia itself. The finding of delirium impact on dementia incidence deserves a separate dissertation. In our cohort, despite the small statistical power due to the few delirium cases detected, history of a delirium episode increased the likelihood of dementia incidence by 8-fold. Moreover, the true impact of delirium in terms of PAF could be underestimated, since our variable is based on participants self-report. Indeed, it is known that delirium is frequently under-recognized in clinical practice [28], with low agreement on diagnosis even among delirium experts [29]. The tight and bidirectional relationship between delirium and dementia is widely reported, while the pathophysiological mechanism underlying this interplay is still poorly understood [5]. However, several preventive interventions are recommended to reduce the risk of delirium in hospitalized older adults, which should be easily implemented in clinical practice [30].

Regarding the other modifiable factors, trends toward significance were found for low education and physical inactivity. However, when all the relevant factors were concurrently tested, the impact of physical inactivity on dementia was reduced. This could be due to the co-presence of other factors in the multivariable model related to cardiovascular health, with possible overlapping effect.

Indeed, low levels of physical activity are an established risk factor for cardiac diseases and diabetes [31], both included in our multivariable model and exerting a significant effect on dementia incidence. It is therefore possible that physical activity act on dementia incidence through an indirect pathway (i.e., preserving cardiovascular health). However, even small amount of physical activity lead to measurable health benefits with low risk of harm and minimal cost [31–33]. Altogether, these findings support to include recommendations on the importance of physical activity for public health, even if the results on the direct effect on cognitive outcomes are not conclusive [33].

No associations were found between late-life hypertension or obesity and dementia, in line with previous evidences consistently showing a significant association with dementia only when these factors are detected in midlife [2, 3, 34, 35]. A counterintuitive finding is the lack of association between age and dementia. This could be due to the specific features of our study, which included by design individuals in a single age quintile at baseline (70–74 years), in order to minimize the confounding effect of age [9].

The discrepancies with previous findings could be also due to the different methodological approach used in the present study. Indeed, previous PAF estimates were calculated on secondary data from studies with heterogeneous designs (meta-analyses, observational, randomized controlled trial), and the co-occurrence of factors was indirectly controlled using communality of factors (i.e., the variance in observed variables accounted for by common factors) calculated on data from national repositories [2, 3, 36]. Conversely, we statistically tested the prospective associations of exposures with disease occurrence on primary data, including all the relevant exposures in the same model. Moreover, we further controlled for non-modifiable factors and for death occurrence within the study period. This led to a more conservative but more realistic estimation of the potential of dementia prevention through risk modification.

To the best of our knowledge, only one previous study investigated all the major modifiable and non-modifiable risk factors on a prospective cohort using a similar methodological approach. It showed an independent impact of APOE ε4, diabetes, depression, low fruit/vegetable consumption, and cognitive reserve (i.e., crystallized intelligence) on dementia or mild cognitive impairment incidence, approaching an overall PAF of 40% for modifiable factors [37]. Therefore, even in a different cohort and measuring a different outcome, APOE ε4 and diabetes were reliably associated with cognitive decline in late life.

The main strength of the present study is the methodological approach, which allows the statistical estimation of the potential of dementia prevention in the real-world setting, concurrently considering all the major modifiable and non-modifiable risk factors within the same population-based cohort. Population-based study controls for selection bias, being therefore particularly appropriate to investigate the potential of prevention through public health interventions addressed to the general population. Moreover, in our study, dementia diagnosis was set after multidimensional assessment and prospectively detected during the follow-up period, fulfilling the temporality criteria between the exposures and the outcome [38].

The main limitation of the study is the relatively low number of dementia cases, leading to reduced statistical power for the less prevalent risk factors. Moreover, since PAF is affected by the frequency of the risk factor in the population of interest, the generalizability of our results is limited to populations with the same key features of the InveCe.Ab cohort. Finally, the operationalization of some risk factors in the present study was not optimal, potentially reducing the sensitivity to detect the risk condition in a portion of cases. For example, hearing loss was evaluated by the clinicians instead of audiometry, loneliness was measured by a single yes/no question, and history of delirium was based on self-report.

## Conclusions

Our results show an independent influence of APOE ε4, diabetes, heart disease, stroke, and delirium on dementia incidence across 8 years in older adults aged between 70 and 74 years. Focused and personalized preventive intervention addressed to these clinical populations and embedded in clinical practice could be an effective approach potentially leading to a 40% reduction in late-life dementia onset, controlling for death within the observation period and for the influence of non-modifiable factors. Further studies on the potential for dementia prevention in diverse populations are needed, since globally the number of dementia cases are expected to exponentially increase particularly in low- and middle-income countries [39].

## Supplementary information

**Additional file 1.** Proportional sub-distribution hazards assessment for diagnosis of dementia. Description of data: assumption of proportionality of hazards for both univariable and multivariable models.

## Data Availability

The datasets used and analyzed during the current study are available from the corresponding author on reasonable request.

## Abbreviations

BMI: Body mass index
DSM IV-TR: Diagnostic and Statistical Manual of Mental Disorders IV
GDS: Geriatric Depression Scale
PAF: Population attributable fraction
SHR: Sub-hazard ratios
