# Supplementary material for: Estimating the potential for dementia prevention through modifiable risk factors elimination in the real-world setting: a population-based study
Source: Alzheimers Res Ther. 2020 Aug 7;12:94. doi: 10.1186/s13195-020-00661-y (PMC7414752; doi:10.1186/s13195-020-00661-y)
Supplement: Supplementary file 1 — Additional file 1. Proportional sub-distribution hazards assessment for diagnosis of dementia. Description of data: assumption of proportionality of hazards for both univariable and multivariable models. [file 13195_2020_661_MOESM1_ESM.pdf]

Additional Table 1: Proportional sub-distribution hazards assessment for diagnosis of dementia, univariable models.

	Main		Time-varying covariates	
**Risk factors**	**Coeff. (95% CI)**	**p value**	**Coeff. (95% CI)**	**p value**
**Non-modifiable**				
Age (class 1935-37)	1.18 (0.12 - 2.23)	0.029	-0.01 (-0.03 - 0.00)	0.058
Sex (female)	-0.23 (-1.15 - 0.70)	0.629	0.00 (-0.01 - 0.01)	0.952
APOE ε4	1.10 (0.12 - 2.08)	0.028	-0.01 (-0.02 - 0.01)	0.385
**Modifiable**				
Low education	0.22 (-0.74 - 1.17)	0.657	0.00 (-0.01 - 0.01)	0.816
Obesity	-0.08 (-1.12 - 0.95)	0.972	0.01 (-0.01 - 0.01)	0.731
Hypertension	0.52 (-0.70 - 1.73)	0.406	-0.01 (-0.02 - 0.01)	0.625
Diabetes	0.65 (-0.32 - 1.61)	0.188	-0.01 (-0.02 - 0.01)	0.635
Depression	-0.11 (-1.97 - 1.75)	0.909	0.01 (-0.02 - 0.03)	0.906
Smoking	0.14 (-1.49 - 1.76)	0.868	0.00 (-0.02 - 0.02)	0.977
Physical inactivity	1.74 (0.71 - 2.77)	0.001	0.02 (-0.01 - 0.01)	0.056
Hearing loss	0.20 (-1.17 - 1.57)	0.779	0.00 (-0.02 - 0.02)	0.983
Loneliness	0.82 (-0.49 - 2.14)	0.218	-0.02 (-0.04 - 0.03)	0.107
Heart disease	0.99 (0.06 - 1.93)	0.038	-0.01 (-0.02 - 0.00)	0.231
Stroke	0.49 (-0.93 - 1.91)	0.498	0.01 (-0.01 - 0.03)	0.435
Head injury	-0.65 (-2.61 - 1.32)	0.519	0.01 (-0.02 - 0.03)	0.600
Delirium	2.88 (1.43 - 4.33)	0.000	-0.01 (-0.04 - 0.01)	0.304

Additional Table 2: Proportional sub-distribution hazards assessment for diagnosis of dementia, multivariable model.

	Main		Time-varying covariates	
**Risk factors**	**Coeff. (95% CI)**	**p value**	**Coeff. (95% CI)**	**p value**
APOE ε4	1.02 (-0-0.3 – 2.08)	0.057	-0.01 (-0.02 – 0.01)	0.399
Low education	0.30 (-0.70 – 1.30)	0.556	0.01 (-0.01 – 0.01)	0.913
Diabetes	0.57 (-0.38 – 1.53)	0.238	0.00 (-0.02 – 0.01)	0.737
Physical inactivity	1.54 (0.49 – 2.59)	0.004	0.00 (-0.01 – 0.02)	0.133
Heart disease	1.08 (0.09 – 2.07)	0.033	0.01 (-0.02 – 0.01)	0.177
Stroke	-0.03 (-1.56 – 1.49)	0.966	0.01 (-0.01 – 0.03)	0.223
Delirium	2.66 (0.94 – 4.38)	0.002	-0.01 (-0.02 – 0.03)	0.548
